# Editorial: From clinical trials to real-world data sciences for value in health: access, utilization, and quality

**DOI:** 10.3389/fpubh.2023.1215392

**Published:** 2023-05-31

**Authors:** Xiangxiang Jiang, Gang Lv, Jing Yuan, Kevin Lu

**Affiliations:** ^1^Department of Clinical Pharmacy and Outcomes Sciences, University of South Carolina, Columbia, SC, United States; ^2^Department of General Surgery, The First Medical Center of Chinese People's Liberation Army General Hospital, Beijing, China; ^3^Department of Clinical Pharmacy and Pharmacy Administration, School of Pharmacy, Fudan University, Shanghai, China

**Keywords:** evidence-based medicine, value-based care, clinical trials, real-world data, real-world evidence

Evidence-based medicine (EBM) has made lasting contributions to clinical medicine by establishing a strong scientific foundation for medical practice, creating more refined structures for assessing the quality of evidence, acknowledging the significance of patient values and preferences in making clinical decisions, and devising a reliable approach for producing credible recommendations (1). The goal of EBM is to incorporate “evidence” into medical practice. In accordance with this approach, randomized controlled trials (RCTs) or the systematic review of multiple RCTs are considered to provide stronger evidence than observational studies (2). However, RCTs have their limitations, such as high costs and small samples. Value-based care (VBC), on the other hand, refers to a delivery model in which healthcare providers are rewarded based on patient health outcomes (3). Therefore, VBC requires EBM as a starting point and converts it to patient value-based data so that patients can receive higher quality care than EBM alone (4).

Most recently, there is a growing importance of real-world data (RWD) and real-world evidence (RWE) in healthcare decision-making. RWD refers to data collected from various sources regarding patient health status and healthcare delivery on a routine basis (5). Examples of RWD may include data extracted from electronic health records, medical claims data, and disease or product registries, as well as other sources such as digital health technologies, all of which can provide information on health status. RWE, on the other hand, is the clinical evidence that pertains to the usage, potential benefits, or risks of a medical product, which is obtained through the analysis of RWD.

The extent to which EMB and VBC can be applied to value in health by incorporating various levels of evidence such as RCTs and RWE remains unclear and has not been extensively discussed. Furthermore, there has been limited discussion on the impact of medication use on patient clinical outcomes, access to medication, and the economic burden. The studies compiled on this topic encompass a broad range of research related to EBM and VBC, including bibliometric analysis of medication safety, retrospective study of antimicrobial stewardship program on antibiotic use, cross-sectional and cohort study on dementia, health survey on health utility and occupational diseases, a meta-analysis on short-term efficacy of non-pharmacological interventions, real-world study (RWS) on medical insurance reimbursement policy, and cost-effectiveness analysis of capecitabine maintenance therapy. We expect that the research on this topic or the evidence they provide will play an informative role in the ongoing development and application of EBM and VBC.

Medication safety is a significant concern in healthcare. A mounting challenge in clinical settings is to prevent harm caused by medication errors, particularly for older adults who require ongoing care and are prescribed multiple medications (6, 7). It is important to pinpoint the primary factors that contribute to medication safety and assess the current state of development of key Research Topics. Xie et al. conducted a study to provide an overview of the current status of medication safety for older adults by identifying significant accomplishments, Research Topics, and emerging trends. They found that there has been significant progress in research on medication safety for older adults over the past two decades, with the United States making notable contributions to the field. Current research is focused on polypharmacy, potentially inappropriate medication use, interventions involving pharmacists, and patient experience and perception.

This topic also collected studies regarding the effect of policy implementation in China. The inappropriate use of antibiotics has emerged as a major contributor to the worldwide spread of antimicrobial resistance (8), with China being a notable example (9). To address this issue, antimicrobial stewardship programs, which are evidence-based guidelines, have been developed (10, 11). A study was conducted by Wang et al. to assess the effectiveness of antimicrobial stewardship programs led by pharmacists. The findings of the study indicated that the implementation of antimicrobial stewardship programs is successful in reducing the length of hospital stay, lowering antibiotic consumption and costs, and improving the appropriateness of antimicrobial use. It is the responsibility of the government to provide sustainable formal education for pharmacists and increase funding and staff support to promote antimicrobial stewardship programs. Similarly, Liu Y. et al. conducted an RWS to evaluate the implementation effect of the hepatitis C medical insurance reimbursement policy in China. They found that the medical insurance reimbursement policy for hepatitis C can encourage patients to seek medical treatment actively, increase the use of direct-acting antiviral (DAA) regimens, reduce the financial burden on patients, and enhance the treatment effectiveness of hepatitis C.

Occupational health has significant social and economic values, and health utility is an integral part of occupational health (12, 13). Cost-utility analysis (CUA) is a comprehensive economic evaluation method and has been widely used to inform policies and programs related to occupational health (14, 15). Unfortunately, studies lack in reporting health utility to support the CUA research in occupational health in China. An occupational health survey in a sample of working-age university staff was conducted by Liu X. et al. to determine the health utility of university staff and to identify potential occupational diseases associated with this occupation and evaluate their impact on health. The results showed that the mean health utility was 0.945, with anxiety/depression and pain/discomfort being the most affected domain. Working-age staff in Chinese universities may have lower health utility compared to the general population. Thus, it is important to conduct a CUA using the health utility data to facilitate the implementation of cost-effective programs.

In addition, this Research Topic also contains other related studies. Lu et al. studied the trends in the prevalence of mild cognitive impairment (MCI) and dementia and determined risk factors associated with the early detection of dementia among U.S. middle-aged and older adults using 10-year longitudinal data. They found a decreasing trend in the prevalence of MCI and dementia in the past decade and associated racial/ethnic and gender disparities among U.S. middle-aged and older adults. A network meta-analysis conducted by Shao et al. to compare the potential short-term effects of non-pharmacological interventions (NPIs) on individuals with prehypertension and identify intervention models that could be effective in future community-based management indicated that NPIs can bring short-term blood pressure reduction benefits for prehypertensive patients. Han et al. evaluated the cost-effectiveness of capecitabine as maintenance therapy for patients with metastatic nasopharyngeal carcinoma (mNPC) and found that capecitabine maintenance plus best-supported care was cost-effective for newly diagnosed mNPC.

## Author contributions

XJ, GL, JY, and KL conceived the idea for the editorial and wrote the initial draft. All authors approved the final version of the editorial.
